# Bayesian sample size determination for cost-effectiveness studies with censored data

**DOI:** 10.1371/journal.pone.0190422

**Published:** 2018-01-05

**Authors:** Daniel P. Beavers, James D. Stamey

**Affiliations:** 1 Department of Biostatistical Sciences, Wake Forest School of Medicine, Winston-Salem, NC, United States of America; 2 Department of Statistical Science, Baylor University, Waco, TX, United States of America; Janssen Research and Development, UNITED STATES

## Abstract

Cost-effectiveness models are commonly utilized to determine the combined clinical and economic impact of one treatment compared to another. However, most methods for sample size determination of cost-effectiveness studies assume fully observed costs and effectiveness outcomes, which presents challenges for survival-based studies in which censoring exists. We propose a Bayesian method for the design and analysis of cost-effectiveness data in which costs and effectiveness may be censored, and the sample size is approximated for both power and assurance. We explore two parametric models and demonstrate the flexibility of the approach to accommodate a variety of modifications to study assumptions.

## Introduction

Cost and effectiveness are often modeled jointly in biomedical studies, and there is typically interest in quantities that are functions of both of these outcomes. Comparing treatments on the basis of cost-effectiveness is increasingly important for clinical trials, and recent publications have considered approaches to assess power for cost-effectiveness studies [1–3]. Often such power calculations assume normally distributed costs and effectiveness, which is convenient as it allows for closed form formulae for sample size determination [4–6]. The bivariate normal approach has been utilized in recent methodological work allow for novel study designs, such as the case of large multicenter trials with or without cluster randomization, where approaches such as maximin have been utilized to obtain optimal sample sizes for cost-effectiveness studies [5, 6]. However, for a variety of reasons, observations are often censored, most commonly as a result of the data originating from a survival study. Cost-effectiveness models for survival data have recently been considered [7, 8]. The case of normally distributed costs and effectiveness with censored cost data is discussed in [8]. However, it is well known that costs are often skewed and gamma and log-normal distributions are used. A wide array of cost-effectiveness models and Bayesian analysis of each is described in [9]. One of the models they consider is a Weibull model for effectiveness and a log-normal model for costs. They then extend the log-normal cost model to a non-parametric model. Skewness and censoring in a cardiovascular trial is accounted for in [7]. However, to our knowledge at this point no studies have proposed sample size determination and power estimation for cost-effectiveness studies with right-censored outcomes.

In this paper we seek to provide a simulation-based algorithm for determining the sample size for a cost-effectiveness study for survival data in which both costs and effectiveness are censored. We consider both traditional power calculations and assurance [10], and we utilize the Bayesian paradigm for at least three reasons. First, numerous authors have noted that when data are not normally distributed, the Bayesian approach is preferable due to the flexibility of parametric assumptions [7, 9, 11], and thus a Bayesian design is applicable. Secondly, in most frequentist designs, nuisance parameters are considered known at the design stage. This is true both in cases where a closed form is used or in the approach of [12] where simulation is used. The Bayesian design approach allows prior information to be utilized to adequately account for uncertainty in nuisance parameters, while also accounting for the uncertainty inherent in these estimates. The simulation-based approach has been increasingly utilized for study planning [13, 14]. Finally, the Bayesian approach allows for parameter estimation by sampling directly from the posterior distribution using Markov chain Monte Carlo methods. We focus our interest on the incremental net monetary benefit (INMB) but our method would be equally useful for incremental cost-effectiveness ratio, average cost-effectiveness ratio, or other measures. We make the code we use available in the appendix so that users would be able to make appropriate changes for alternative criteria or parameters of interest.

We focus our sample size determination scheme on both power and assurance. Assurance, which will be more clearly defined in Section 1, has been defined as the unconditional probability that a trial achieves a positive outcome in a statistical sense [10], such as statistical significance, and can be useful to account for uncertainty in outcomes derived from multiple parameters. It can be thought of as expected power over all likely values of a parameter rather than for a fixed value under the alternative hypothesis. It has been applied in a variety of models in clinical trials [10, 15–17], including trials with time-to-event outcomes [18], but we extend this prior work to time-to-event cost-effectiveness trials with censored data.

Our paper is organized as follows: we present the two parametric approaches for analysis in Section 1, and we discuss Bayesian considerations to the design of cost-effectiveness studies in Section 2. In Section 3 we discuss priors for the design stage and the algorithm for the sample size determination. In Section 4 we consider examples to demonstrate the method, utilizing both cost-effectiveness models from Section 1. Finally, we discuss our approach and consider new directions for research in Section 5.

## 1 Methods

Cost-effectiveness models in which effectiveness is measured by survival time are generally defined as the bivariate relationship between survival time, *T*, and treatment cost, *C*. It is of interest to model

$$\begin{array}{c} f\left(t,c\right)=f^{T}\left(t\right)f^{C|T}\left(c|t\right). \end{array}$$ (1)

When censoring occurs survival times are not fully observed, and cost data at the time of censoring underestimate the true cost had survival times been fully observed. Using censored values as replacements for fully observed survival time and costs will lead to biased estimates of cost-effectiveness, and estimation for a model of this complexity makes a parametric form useful, especially if the proportional hazards assumption is restrictive. In particular, a Bayesian approach using Markov chain Monte Carlo (MCMC) estimation can be easily implemented to flexibly model such a scenario. Determining clinical effectiveness in the presence of censored survival times is common. However, accounting for the accrued costs in the presence of incomplete survival time is far less common, complicated by a reliance on cost estimates conditioned on latent time quantities. Fortunately the MCMC approach makes this type of estimation very straightforward. Missing time values are imputed at each iteration of the chain from the conditional posterior distribution, and the imputed observations can be used to estimate the total costs and variability at the estimated failure time.

Here, we apply parametric models for both costs and effectiveness. We proceed under the context of clinical trials, although the results apply generally to any cost-effectiveness study in which treatment alternatives are being compared. We assume the *i*th subject is randomly assigned to one of two protocols such that *z*_*i*_ = 1 for treatment and *z*_*i*_ = 0 for control. Effectiveness is defined based on survival time, which we denote *T*_*i*_, such that incremental treatment effectiveness is *E*(*T*|*Z* = 1) − *E*(*T*|*Z* = 0). Similarly, we define cost as *C*_*i*_, and the incremental treatment cost is *E*(*C*|*Z* = 1) − *E*(*C*|*Z* = 0).

### 1.1 Outcome

Our focus is on the outcome of the incremental net monetary benefit (INMB), which is the average per-patient benefit of treatment where benefit is defined in financial terms [19]. The INMB is a function of the maximum willingness to pay for a unit of health (WTP), which varies based on a payer’s financial ability and willingness. Put simply, the INMB is the price of improved or sustained health (unit of health × WTP) minus the cost. In terms of the variables above, we define INMB as

$$\begin{array}{c} \begin{array}{cc} INMB(WTP)= & [E(T|Z=1)-E(T|Z=0)]\times WTP\\ & -[E(C|Z=1)-E(C|Z=0)]. \end{array} \end{array}$$ (2)

If the INMB is determined to exceed 0 for a given WTP then the new treatment (*z* = 1) is assumed to be cost effective compared to the alternative (*z* = 0). Thus, we are interested in testing the hypotheses *H*_0_: *INMB* = 0 versus *H*_1_: *INMB* > 0, and we conclude that the INMB is significant by requiring that for the posterior distribution of the INMB, *Pr*(*INMB* > 0) ≥ 1 − *ϕ*, where *ϕ* is the probability of falsely concluding *H*_1_ is true. Common values for *ϕ* include 0.05, 0.025, and 0.01.

Therefore, for the purposes of study planning, we seek the total sample size, *n*, to achieve 1) a prespecified posterior power, or probability of a successful trial under the chosen alternative hypothesis, for fixed parameter values, or 2) assurance for variable parameter values. Furthermore, we wish to vary the potential details of the study, such as study length and assumed distribution for *f*(*t*, *c*), as well as incorporate the uncertainty of values such that the resulting power conditions on a range of possible scenarios for both the parameters of the model and values for WTP.

### 1.2 Model 1: The Weibull-gamma cost-effectiveness model

The first model we employ has been described previously [7]. For the joint distribution in (1), *T*_*i*_ > 0 and is assumed to have a Weibull(*α*_*i*_, λ_*i*_) distribution, where the parameterization of the Weibull is

$$\begin{array}{c} f^{T}\left(t\right)=\alpha {\left(t/\lambda \right)}^{\alpha -1}exp\left[-{\left(t/\lambda \right)}^{\alpha }\right]/\lambda . \end{array}$$

Furthermore, *C*_*i*_ conditioned on time is assumed to follow an alternately parameterized gamma(*μ*(*t*_*i*_), *ν*_*i*_) distribution, where *C*_*i*_ > 0 and the pdf of the gamma distribution is

$$\begin{array}{c} f^{C|T}(c|T_{i}=t_{i})={\left(\nu /\mu \left(t_{i}\right)\right)}^{\nu }c^{\nu -1}exp\left[-\left(\nu /\mu \left(t_{i}\right)\right)c\right]/\Gamma \left(\nu \right) \end{array}$$

such that the mean *E*[*C*|*T*_*i*_ = *t*_*i*_] = *μ*(*t*_*i*_) depends on the survival time for the *i*th participant *t*_*i*_, and similarly *Var*[*C*|*T*_*i*_ = *t*_*i*_] = *μ*(*t*_*i*_)^2^/*ν*.

We allow for each randomization arm to have unique parameters, allowing for flexibility in both effectiveness and cost models. For the survival/effectiveness model, we define shape parameter

$$\begin{array}{c} \alpha_{i}=\left(1-z_{i}\right)\alpha_{0}+z_{i}\alpha_{1} \end{array}$$

and scale parameter

$$\begin{array}{c} \lambda_{i}=\left(1-z_{i}\right)\lambda_{0}+z_{i}\lambda_{1}, \end{array}$$

where *α*_*i*_ > 0 and λ_*i*_ > 0. We define the expected cost of treatment as being linearly related to survival time, such that the conditional mean cost

$$\begin{array}{c} \mu \left(T_{i}\right)=[\beta_{0}+\gamma_{0}T_{i}]\left(1-z_{i}\right)+[\beta_{1}+\gamma_{1}T_{i}]z_{i}, \end{array}$$

and shape parameter

$$\begin{array}{c} \nu_{i}=\left(1-z_{i}\right)\nu_{0}+z_{i}\nu_{1} \end{array}$$

where *ν*_*i*_ > 0. We constrain *μ*(*T*_*i*_) > 0 by defining *β*_*k*_ > 0 and *γ*_*k*_ > 0 where *k* ∈ {0, 1}. This somewhat unusual constraint allows us to interpret the intercept as the “start-up costs” of treatment, and the slope as the average cost accrual of treatment per unit time (ie month, year, etc). Costs can only accrue in a nonnegative direction as time increases.

Using this definition, (2) is redefined as

$$\begin{array}{c} \begin{array}{c} INMB(WTP)=[\lambda_{1}\Gamma \left(1+1/\alpha_{1}\right)-\lambda_{0}\Gamma \left(1+1/\alpha_{0}\right)]\times WTP\\ -[\beta_{1}+\gamma_{1}\lambda_{1}\Gamma \left(1+1/\alpha_{1}\right)-\beta_{0}-\gamma_{0}\lambda_{0}\Gamma \left(1+1/\alpha_{0}\right)]. \end{array} \end{array}$$ (3)

Our approach uses the gamma distribution to model right-skewed costs, but other distributions have been employed successfully as well, including the lognormal and inverse-gamma distributions [9, 20]. Generally it is suggested that sensitivity analyses be performed to consider the robustness of the results to alternate parameterizations of costs [11]. The choice of prior distributions for survival and cost model parameters depends on the available knowledge at the study origin. In the absence of information, we assume diffuse gamma prior distributions on all parameters as elaborated in Section 4.

### 1.3 Model 2: The normal-normal cost-effectiveness model

An alternate parameterization utilizes the bivariate normal distribution for cost-effectiveness, similar to that published in sample size determination methods for cluster-randomized and multicenter cost-effectiveness trials [5, 6]. Although costs and effectiveness are often non-normal, the convenience of modeling and interpretability has made the bivariate normal a common and robust tool for assessing cost-effectiveness [21], particularly for large trials where asymptotic properties can be invoked [22]. Furthermore, parameters are much more easily interpreted, and Bayesian approaches that rely on MCMC methods for estimation are faster. We extend the model of [8] to allow for censored costs and effectiveness. In doing so, we again utilize (1), so that we begin with *f*(*t*, *c*) = *f*(*t*)*f*(*c*|*t*) where

$$\begin{array}{c} T_{i}∼Normal\left(\mu_{T_{i}},\tau_{T_{i}}^{2}\right) \end{array}$$

where

$$\begin{array}{c} \mu_{T_{i}}=\mu_{0}\left(1-z_{i}\right)+\mu_{1}z_{i} \end{array}$$

and

$$\begin{array}{c} \tau_{T_{i}}^{2}=\tau_{T_{0}}^{2}\left(1-z_{i}\right)+\tau_{T_{1}}^{2}z_{i}. \end{array}$$

Next, the distribution of costs given *T*_*i*_ = *t*_*i*_ is

$$\begin{array}{c} C_{i}|T_{i}∼Normal\left(\mu_{C_{i}|T_{i}},\tau_{C_{i}}^{2}\right) \end{array}$$

where

$$\begin{array}{c} \mu_{C_{i}|T_{i}}=\left(\theta_{1}+\theta_{2}t_{i}\right)\left(1-z_{i}\right)+\left(\theta_{3}+\theta_{4}t_{i}\right)z_{i} \end{array}$$

and

$$\begin{array}{c} \tau_{C_{i}}^{2}=\tau_{C_{0}}^{2}\left(1-z_{i}\right)+\tau_{C_{1}}^{2}z_{i}. \end{array}$$

Eq (2) is thus redefined as

$$\begin{array}{c} INMB\left(WTP\right)=[\mu_{1}-\mu_{0}]WTP-[\theta_{3}+\theta_{4}\mu_{1}-\theta_{1}-\theta_{2}\mu_{0}]. \end{array}$$ (4)

The use of the normal-normal model assumes relatively bell-shaped and symmetric bivariate distributions for survival times and costs. Users of the model should carefully inspect data to ensure this is a justifiable assumption, with the added caveat that in our scenario we might have right-censored data. Furthermore, poorly chosen simulation parameters could yield negative survival times or costs, so in our programs we omit negative values and resample the observation in the rare event of negative times or costs. We assume diffuse normal prior distributions for *μ*_0_, *μ*_1_, *θ*_1_, *θ*_2_, *θ*_3_, and *θ*_4_ and diffuse inverse gamma distributions for $\tau_{T0}^{2}$, $\tau_{T1}^{2}$, $\tau_{C0}^{2}$, and $\tau_{C1}^{2}$. Further information is presented in Section 4.

### 1.4 Data likelihood

As previously presented by [7], if we define the death indicator for the *i*th participant *d*_*i*_ and *t*_*i*_ the minimum of the censoring or survival time, then the log-likelihood is the sum of

$$\begin{array}{c} L_{i}=d_{i}[lnf^{T}\left(t_{i}\right)+lnf^{C|T}\left(c_{i}|t_{i}\right)]+\left(1-d_{i}\right)ln\int_{t_{i}}^{\infty }f^{T}\left(u\right)(\int_{c_{i}}^{\infty }f^{C|T}\left(x|u\right)dx)du. \end{array}$$

The normal-normal model has a similar structure, except with different underlying distributions for the time and cost models.

### 1.5 Assurance

Traditional statistical power is an estimate of the probability of rejecting a null hypothesis under a fixed alternative hypothesis, which in a clinical trial is usually the treatment effect. Assurance therefore is the probability of rejecting a null hypothesis under the *distribution* of likely values of a treatment effect. Therefore, the assurance is frequently described as the unconditional power of the desired outcome because the probability of a trial success (typically defined as rejection of the null hypothesis based on external criteria) does not assume a fixed alternative hypothesis value [18]. Assurance is often estimated using Monte Carlo simulations to draw a random value for the treatment effect its distribution at each iteration, which will be explained in detail in Sections 2 and 3. For the cost-effectiveness case, we will need to consider both cost and effectiveness for assurance estimates. While the approach does not necessarily require a Bayesian data analysis, a Bayesian approach helps with the estimation of INMB under censored observations.

## 2 Bayesian design

The sample size/power determination algorithm approach of Wang and Gelfand requires two sets of prior distributions. The first set is referred to as the design (or sampling) prior. This prior is generally concentrated on the part of the parameter space where interest lies, and it is used to generate data in the simulation algorithm to determine the model’s operating characteristics such as power. The second prior is the analysis (or fitting) prior and is used in the data analysis of each simulation-generated data set. Typically, these are also used in the final analysis when the actual data is collected. The design and analysis priors can be the same, though often the analysis priors will be less informative than the design priors.

A common concern in Bayesian analyses regards the informativeness of the prior distributions, in this case the analysis prior, particularly regarding the level of information required to be known prior to the conduct of the study. If prior information regarding the parameters is available, this can easily be represented in the analysis prior distribution. However, if concerns that subjective or biased information could guide the analytic results of the study due to overly influential prior information, noninformative priors should be sufficient to minimize most concerns. In the current approach, we utilize noninformative priors in the sense that they are typically assigned means in line with the null hypothesis (i.e. zeros for regression parameters) and with large variances to allow the data likelihood to dominate the posterior distribution, comparable to a frequentist approach. Nothing about the method prohibits or demands noninformative priors; this choice is available to the analyst, and full disclosure should be provided whichever approach is chosen. The model is relatively robust to misspecification of priors when using noninformative priors, but a misspecified informative prior could lead to posterior means that are overly influenced or restricted by incorrect prior information.

Traditional sample size determination methods typically rely on fixed quantities rather than design prior distributions based on available evidence. The use of design prior distributions allows for researchers to represent uncertainty about the prior evidence for the parameters; furthermore, the traditional approach can be considered a special case of our proposed method in which the design prior distribution has zero variance and all probability mass on the chosen fixed quantity. The use of fixed values, while a common practice, may lead to overstatement of model power due to an investigator being forced to select a value that is unlikely to be observed in the actual study. We consider both cases here in order to directly compare the differences. Our approach allows for more uncertainty, and in Section 4 we demonstrate the difference between assurance, where we use a flexible design prior distribution, compared to posterior power, where we use a fixed values for the design priors (i.e. zero variance distributions).

We utilize a simulation-based approach to determine the required sample size [12]. We denote the data vector for a sample size *n* as **d**^*n*^ = (**t**, **c**, **z**), where **t** = (*t*_1_, …, *t*_*n*_), **c** = (*c*_1_, …, *c*_*n*_), and treatment indicator vector **z** = (*z*_1_, …, *z*_*n*_). We denote the vector of parameters *θ* = (*α*_0_, λ_0_, *ν*_0_, *β*_0_, *γ*_0_, *α*_1_, λ_1_, *ν*_1_, *β*_1_, *γ*_1_) for the Weibull-gamma model and *θ* = (*μ*_0_, *τ*_*T*0_, *θ*_1_, *θ*_2_, *τ*_*C*0_, *μ*_1_, *τ*_*T*1_, *θ*_3_, *θ*_4_, *τ*_*C*1_) for the normal-normal model.

One important assumption that is particularly applicable to the design phase that is easily overlooked is the choice of a censoring distribution. Previous literature has demonstrated the censoring distribution can affect the power of a chosen model [23, 24], and although inference on the censoring distribution is rarely performed during analyses, it is crucial for the censoring distribution to be realistic and relatively robust to the analytical approach. This is particularly important because censored costs are estimated based on the data augmented survival times. Censoring distributions with higher variances can lead to higher variability in INMB estimates, resulting in loss of power. This is considered in Section 4.3.

Statistical assurance is the unconditional probability that a trial will yield a specified outcome, often success [9]. We distinguish this from power by noting that assurance is the expected power with respect to the design prior. For the present problem note that INMB is a function of all model parameters. We seek a sample size *n* for our study, incorporating uncertainty about the paramter values, that yields an effect size of INMB that is desired to detect success (*Pr*(*INMB* > 0)) with power *π*(*θ*). The power function is then defined as *π*(*θ*) = *P*(*R*|*θ*) where *R* denotes rejection of the hypothesis. Assurance is then the unconditional probability of rejection

$$\begin{array}{c} P\left(R\right)=E(\pi (\theta )), \end{array}$$

where this expectation is with respect to the design priors. Unlike power which generally goes to 1 as the sample size increases, assurance is dependent on the design prior, and depending on the informativeness and location of the sampling prior, the maximum assurance that a model can attain is less than 1.

## 3 Description of sample size determination algorithm

The sample size determination method uses a Monte Carlo simulation approach to generate data randomly selected from the design priors for a fixed *n* and to analyze the data using the proposed model. This is repeated for multiple sample sizes and in most cases the smallest sample size for which some optimality criteria has been achieved is selected as the preferred sample size. In our case, we focus on the probability that the INMB for our active treatment exceeds the control at a statistically significant threshold, such as 95% posterior probability, at a prespecified WTP value, *Pr*(*INMB* > 0|*WTP*) > 0.95. In our simulation, we would look for this threshold to be met or exceeded 80% or more for datasets generated from our design priors. The smallest sample size that meets these conditions will be chosen, as detailed below.

Prior to beginning the algorithm, specify the design priors, analysis priors, censoring distribution, and the WTP values of interest. Furthermore, being a simulation-based algorithm, a reasonable set of possible sample sizes should be preidentified to explore the properties of the model given *n*. Then, execute the following steps for the sample size determination algorithm.

1. Generate values for *θ* from the design priors. This is particularly important for assurance, but when the desired outcome is power, the parameters are usually fixed.
2. For a reasonable sample size *n*, generate values for **t** and **c** of length *n* from the distributions with *θ* generated in step 1. Generate censored observations as appropriate, and when censoring exists, segregate censored observations for time and cost from the fully observed times and costs.
3. Fit the Bayesian model defined by Eqs 3 and 4 to the simulated data generated in steps 1 and 2 using the analysis priors and approximate the posterior distribution of the INMB, particularly the appropriate alternative hypothesis posterior probability *Pr*(*INMB* > 0|*WTP*) > 0.95 for each WTP value. Code for fitting each of the models discussed in the paper in WinBUGS/OpenBUGS are presented in the Appendix.
4. Repeat steps 1–3 *B* times at each sample size value under consideration.
5. Calculate the probability of rejecting the null hypothesis *r*^(*n*)^ by calculating the proportion of the *B* iterations where the posterior *Pr*(*INMB* > 0|*WTP*) > 0.95 via the formula
  $$\begin{array}{c} r^{\left(n\right)}=1/B\sum_{k=1}^{B}I\{Pr\left(INMB>0|WTP,d^{\left(n,k\right)}\right)>0.95\}. \end{array}$$
  When the design priors are fixed, *r*^(*n*)^ is equal to the power, and when the design priors are variable, this proportion is the assurance.
6. Finally, repeat steps 1–5 for a range of sample sizes and plot *r*^(*n*)^ by WTP and *n* to find a sample size that achieves a desired level of power or assurance. We approach this two ways in our examples.

In our case we wish to obtain n* such that *Pr*(*Pr*(*INMB* > 0|n*) ≥ 0.95) ≥ 0.8; that is, at least 80% of simulated data sets yield an INMB with at least 95% posterior probability density greater than 0, which is roughly analogous to 80% power or assurance. Our choice for *B* was 300, although reduced Monte Carlo error can be achieved at with higher values of *B*, at the expense of time. Alternate values of posterior probability than 0.95 can easily be substituted in steps 3, 5, and 6.

## 4 Results

As a demonstration of the method, we organize the results section as follows: in Section 4.1 we assume the data arise from the Weibull-gamma model using fixed quantities for design parameters, which allows for estimation of posterior power; in Section 4.2 we assume the data arise from the Weibull-gamma model using variable quantities for design parameters, which allows the determination of assurance; in Section 4.3 we modify the assumed censoring distribution to determine the impact of an altered censoring distribution on model power; and finally, in Section 4.4 we modify the parametric form of the study to explore the normal-normal cost-effectiveness model for both design and analysis.

We generated 300 iterated data sets for each sample size using R v2.12.2, and we fit the data in WinBUGS using the R2WinBUGS library. For each data set, we generated 3 chains using a 2000 iteration burn-in and keeping a 10, 000 iteration sample. Of primary interest was *Pr*(*INMB* > 0|**c**, **t**) for an array of different WTP quantities ranging from $100k to $350k by $50k increments. The following examples demonstrate the performance of the method, and we further show how modifications to the study design impact the power and sample size, including the duration of the study and the assumed censoring distribution. The proposed method is extremely flexible and, being simulation-based, is far more adaptable to a variety of inputs than standard closed-form methods. The WinBUGS/OpenBUGS code is available in the appendix as S1 and S2 Files, while the R programs used to simulate the data are presented as appendix S3 and S4 Files.

### 4.1 Bayesian power using the Weibull-gamma cost-effectiveness model

For the determination of power, we assume fixed quantities for the design priors. We present the chosen parameter values in Table 1 for both the design and analysis portions. We consider two potential lengths for the trial; first, we assume that censoring times are distributed uniformly from 2.5 to 3.5 years, which would be realistic for a study with a 12-month recruitment period that accrues survival times for an average of 3 years with a maximum of approximately 3.5 years. The second scenario assumes that censoring times are distributed uniformly from 3.5 to 4.5 years, which is realistic for a study with a 12-month recruitment period that accrues survival times for an average of 4 years and no more than 4.5 years. It is expected that a longer study will allow for a more precise estimate of INMB due to fewer censored observations, although whether the added effort yields superior power is answered by the simulation results. These same parameter values are used for the first three examples, and based on the chosen parameter values in each case the benefit expectation *E*[*INMB*|*WTP*] = 1.93 × *WTP* − 150.

**Table 1:** Design and analysis prior distributions for Weibull-gamma models in Sections 4.1, 4.2, and 4.3.

Parameters	Power Design Priors	Assurance Design Priors	Analysis Prior Distribution
*α*_0_	0.75	*normal*(0.75, 0.1^2^)	*gamma*(0.1, 0.1)
λ_0_	0.9	*normal*(0.9, 0.1^2^)	*gamma*(0.1, 0.1)
*ν*_0_	1.2	*normal*(1.2, 0.15^2^)	*gamma*(0.1, 0.1)
*α*_1_	0.5	*normal*(0.5, 0.05^2^)	*gamma*(0.1, 0.1)
λ_1_	1.5	*normal*(1.5, 0.2^2^)	*gamma*(0.1, 0.1)
*ν*_1_	3	*normal*(3, 0.2^2^)	*gamma*(0.1, 0.1)
*β*_0_	50	*normal*(50, 10^2^)	*gamma*(10, 0.1)
*γ*_0_	75	*normal*(75, 15^2^)	*gamma*(10, 0.1)
*β*_1_	100	*normal*(100, 18^2^)	*gamma*(10, 0.1)
*γ*_1_	60	*normal*(60, 15^2^)	*gamma*(10, 0.1)

**Fig 1:**
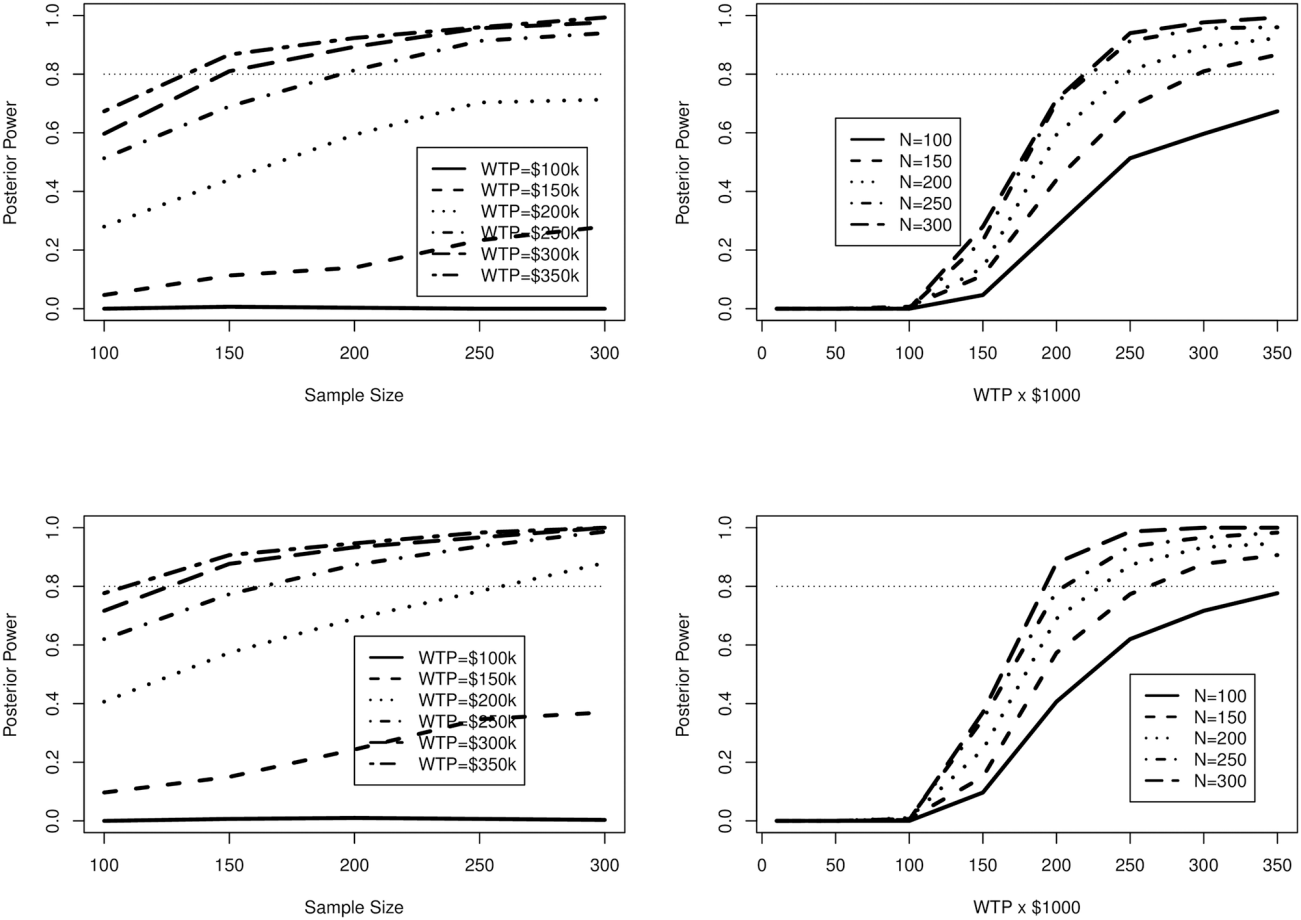
Posterior power estimates for Section 4.1. Average censoring time is 3 years for upper figures and 4 years for lower.

In Fig 1 we present the posterior power for detecting *INMB* > 0 presented as functions of sample size grouped by WTP values and alternately as WTP values grouped by sample size. We observe that the power for a trial of longer duration yields higher posterior power due to the reduced proportion of censored observations, and that the chosen WTP can have a very large impact on the preferred sample size, with larger WTP values requiring smaller sample sizes to achieve 80% power. However, we know that these values could potentially overstate our knowledge of the randomization effect, particularly if prior data are ambiguous or absent. We feel it is appropriate to place less stringent assumptions on our knowledge of cost-effectiveness by contrasting these results to those of an assurance analysis.

### 4.2 Bayesian assurance using the Weibull-gamma cost-effectiveness model

In the second example we modify the previous simulation except we utilize the variable design priors to provide estimates of assurance for which traditional fixed value inputs are utilized. Estimation of assurance can furthermore demonstrate the inflation in power if we naïvely assume fixed values are “correct” rather than providing variable quantities to capture our uncertainty about the true state of nature. For the subsequent output, we repeat the scenarios from Section 4.1 with the sole exception that at each iteration of the program, we generate a design prior value for each parameter from the distributions indicated in the “Assurance Design Priors” column of Table 1.

**Fig 2:**
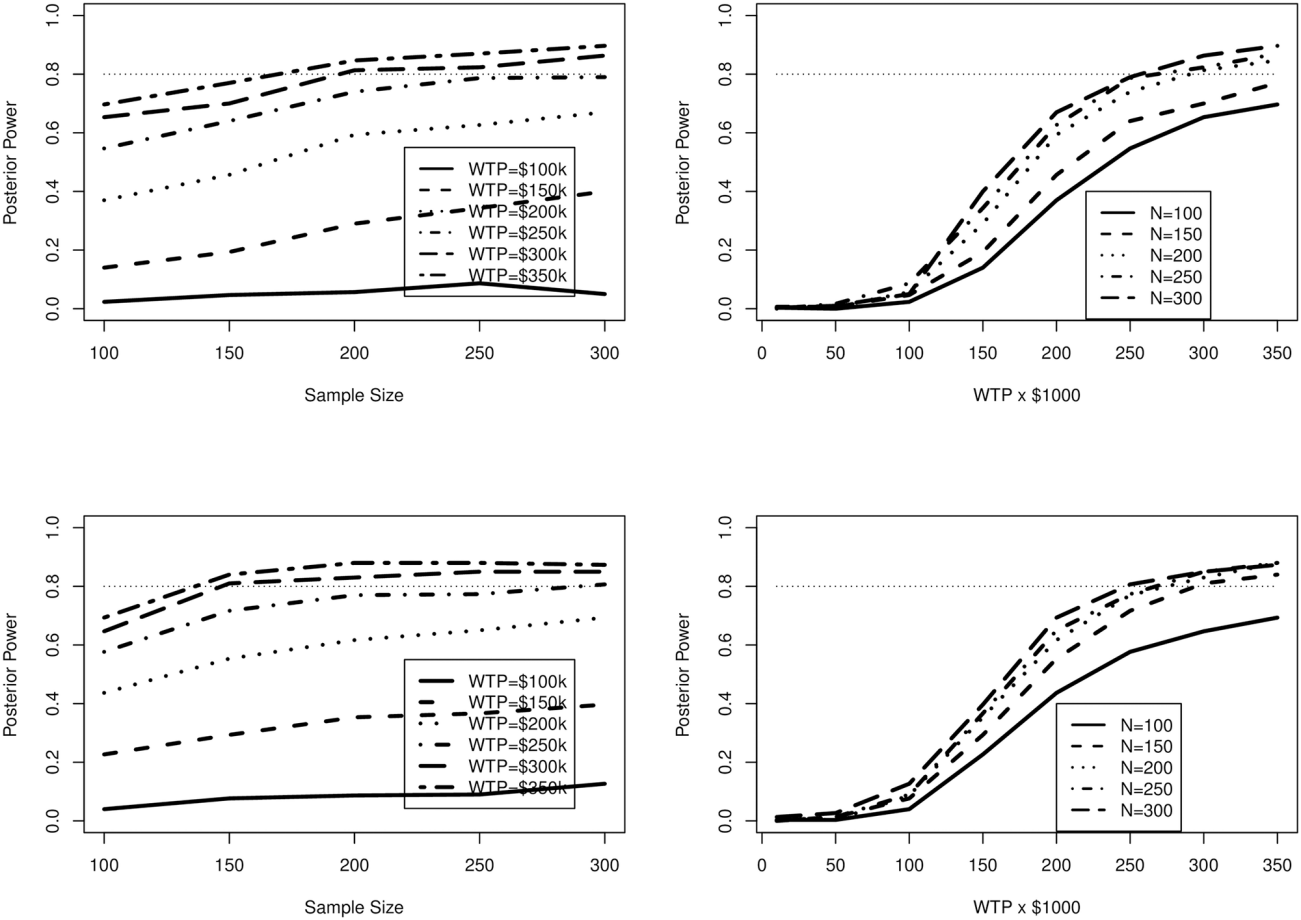
Posterior assurance estimates for Section 4.2. Average censoring time is 3 years for upper figures and 4 years for lower.

We observe the posterior assurance output in Fig 2. There is a marked reduction in posterior assurance compared to the power output in Fig 1. Importantly, we can quantify the penalty for using more stringent assumptions in Section 4.1. For example, in the upper half of Fig 2, corresponding to a trial of 3 years, the power for $250k WTP at *n* = 200 is approximately 70%, while in Fig 1, the posterior power is approximately 80%. Our added uncertainty about the true value of the input parameters that combine to produce the INMB has led to a 10% reduction in the probability a successful trial, and to achieve 80% assurance, a larger sample size is required than for 80% power. Furthermore, we note that in Fig 1 the curves clearly asymptotically approach 1.0, while in Fig 2 the asymptotic trend seems to be toward a value somewhat less than 1.0.

### 4.3 Effects of modified censoring distribution on Weibull-gamma power

We modify the simulation in Section 4.1 to allow for a different censoring function. Most survival studies experience censored observations due to the study conclusion and due to non-death attrition prior to the study termination. To accomodate this, we allow for a minority of participants to withdraw from the study prior to the termination of the study. In our case, for each participant, we generate a binary indicator with probability 0.8 such that a 1 indicates the censoring value is generated as described in Section 4.1 and a 0 indicates the participant was participant’s censoring distribution is randomly sampled from a uniform distribution from 0 to 2.5 or 3.5, depending on whether the mean study duration is 3 years or 4 years, respectively. If the observed death time is greater than the generated censoring time, then the participant is considered censored at the censoring time. This is a far more variable censoring distribution than in Section 4.1; it is much more likely to censor individuals at relatively small follow-up times (i.e. *T* < 1 yr). The net result is that we can compare the power to that of Section 4.1 and determine how sensitive our model is to the assumptions governing the censoring distribution.

**Fig 3:**
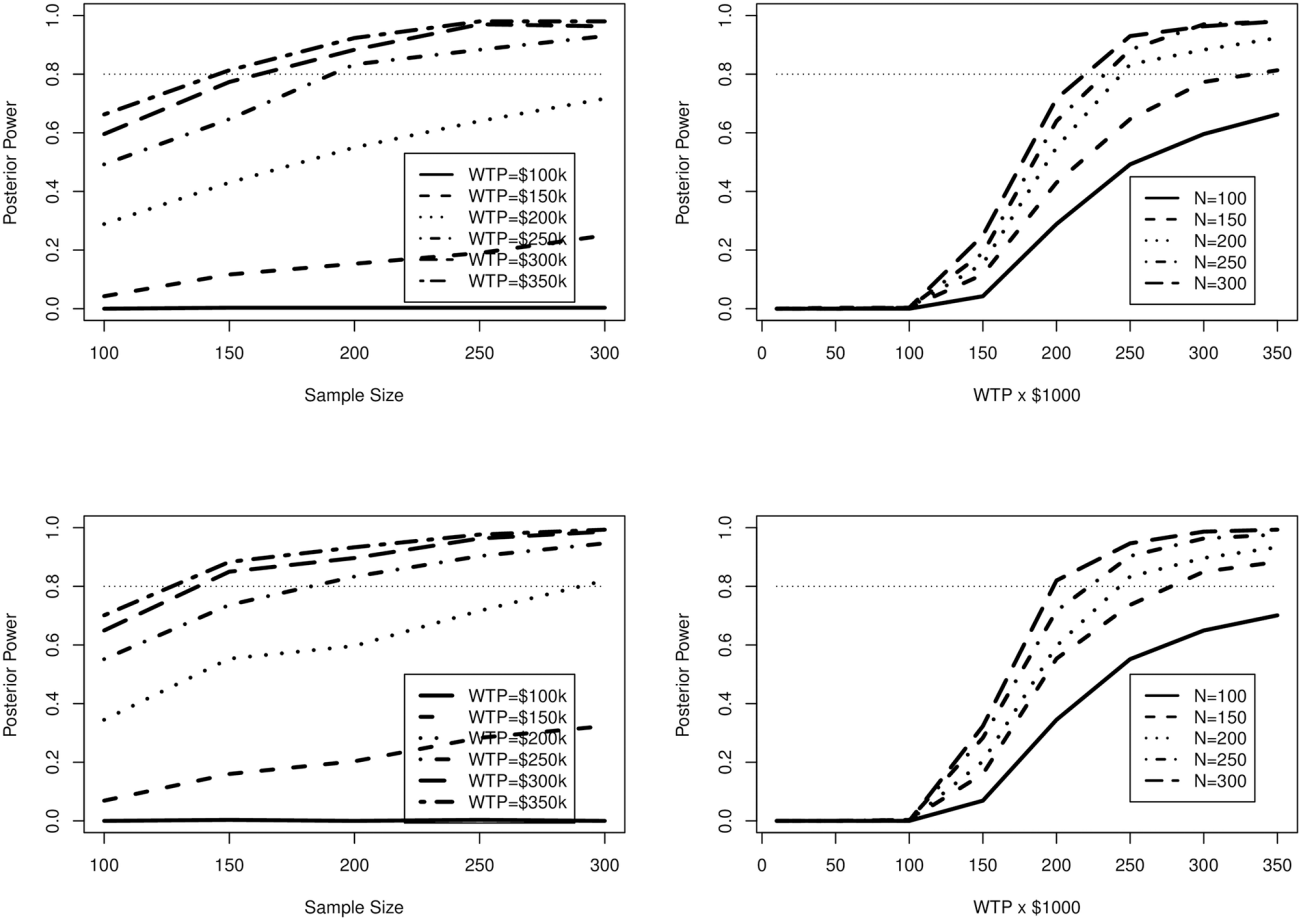
Posterior power estimates from Section 4.3. Average study duration time is 3 years for upper figures and 4 years for lower.

In both the cases for the 3 year proposed study and 4 year proposed study, as seen in Fig 3, the added variability of the exponential censoring function slightly reduces the power compared to the censoring mechanism in Section 4.1. This is important because all other components are the same; increased variability in censoring times lead to reduced power. While this finding is expected, we can quantify the impact of different censoring types; for studies in which participants may drop from participation prior to the completion of the follow-up time, we can quantify the difference this type of censoring as opposed to the type defined in Section 4.1.

### 4.4 Bayesian power using the normal-normal cost-effectiveness model

The final approach assumes that costs and survival times are distributed bivariate normal as proposed in Section 1.3, except in this case time is defined as months rather than years. The outcome of interest is Bayesian power or sample size, and thus design priors are assumed to be fixed quantities. The design and analysis priors are presented in Table 2, which corresponds to a bivariate normal distribution for treatment *Z* = 0 of

$$\begin{array}{c} \left(\begin{array}{c} T_{0}\\ C_{0} \end{array})∼N((\begin{array}{c} 33.5\\ 700 \end{array}),(\begin{array}{cc} 8.5^{2} & 0.4(8.5)(101.5)\\ 0.4(8.5)(101.5) & 101.5^{2} \end{array})\right) \end{array}$$

and for treatment *Z* = 1

$$\begin{array}{c} (\begin{array}{c} T_{1}\\ C_{1} \end{array})∼N((\begin{array}{c} 25.0\\ 130 \end{array}),(\begin{array}{cc} 8.5^{2} & 0.3(8.5)(30)\\ 0.3(8.5)(30) & 30^{2} \end{array})). \end{array}$$

The values for the cost model parameters in Table 2 are conditional on *T*. For the fourth example, based on the chosen parameter values the benefit expectation *E*[*INMB*|*WTP*] = 8.5 × *WTP* − 570.

**Table 2:** Design and analysis prior distributions for normal-normal model in Sections 4.4.

Parameters	Power Design Prior Values	Analysis Prior Distribution
*μ*_0_	25.0	normal(0, 10 000)
$\tau_{T0}^{2}$	8.5^2^	InvGamma(0.01, 0.01)
*μ*_1_	33.5	normal(0, 10 000)
$\tau_{T1}^{2}$	8.5^2^	InvGamma(0.01, 0.01)
*θ*_1_	103.5	normal(0, 10 000)
*θ*_2_	1.06	normal(0, 10 000)
$\tau_{C0}^{2}$	28.6^2^	InvGamma(0.001, 0.001)
*θ*_3_	539.9	normal(0, 10 000)
*θ*_4_	4.78	normal(0, 10 000)
$\tau_{C1}^{2}$	93.0^2^	InvGamma(0.001, 0.001)

**Fig 4:**
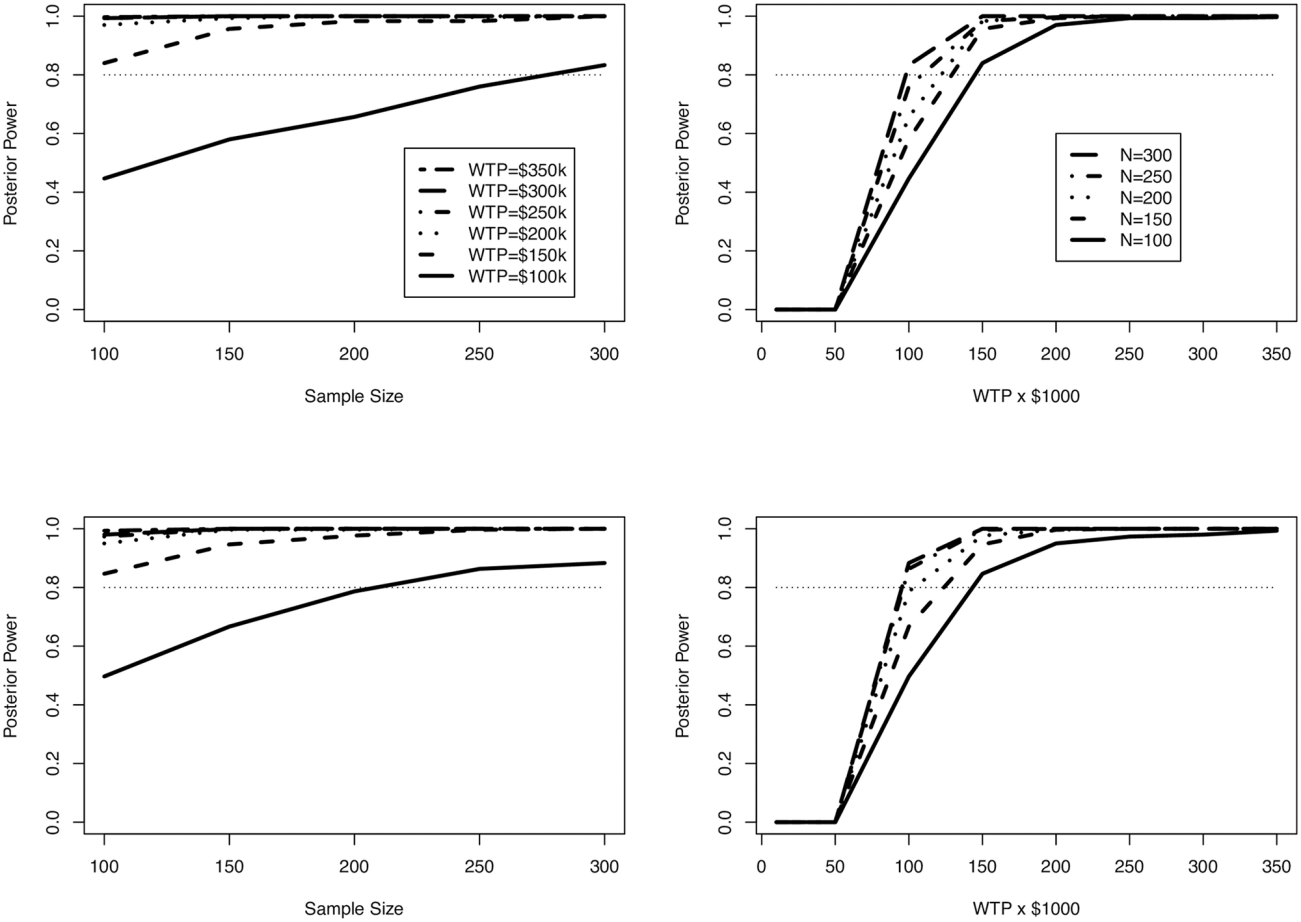
Posterior power estimates from Section 4.4. Average censoring time is 3 years for upper figures and 4 years for lower.

For this simulation, we generate censoring values from a normal distribution with mean 36 months and standard deviation 2.4 for the top row of plot in Fig 4 and with mean 48 and standard deviation 2.4 for the bottom row, corresponding to censoring times of an average of 3 years and 4 years, respectively (although the data were generated with survival in months rather than years). We observe the prior information used in both the design and analysis phase in Table 2. While a direct comparison with the Weibull-gamma approach is not valid due to the differing parameterization of this model compared to preceding sections, the parameters were selected to at least mimic the previous models, and the data were generated ensuring no negative values existed for time or costs.

## 5 Discussion

The Bayesian power and sample-size determination approach for cost-effectiveness studies with censored data offers key advantages over existing cost-effectiveness power calculation methods. Users can represent unknown quantities as design prior distributions with variances rather than fixed quantities; survival times and costs can be fully simulated to calculate the INMB rather than relying on previous INMB estimates; and the method is extremely flexible and adaptable for a variety of distributions and assumptions, utilizing freely available statistical software packages.

The motivating example was inspired by clinical trials in which there is a high death rate, such as cancer or heart failure. Subsequent applications in which there is a lower censoring rate should monitor the imputed observations to ensure the distributional assumptions do not yield unrealistic estimates. Most participants expired prior to censoring in our motivating examples, but in scenarios in which both event times and censoring times can be highly variable, such as incident cardiovascular disease, WinBUGS or OpenBUGS can easily provide posterior estimates of the censored participants’ failure time distributions left-truncated at the censoring time. Furthermore, the current work focuses on simple parallel randomized controlled trials, but future work should consider alternate design approaches including cluster randomized trials and minmax designs.

## Supporting information

S1 File The first OpenBUGS program was used to produce INMB estimates for the gamma-Weibull cost-effectiveness model. (TXT)

S2 File The second OpenBUGS program was used to produce INMB estimates for the normal-normal cost-effectiveness model. (TXT)

S3 File The wg_simulation.r program was used to simulate data for the Weibull-gamma cost effectiveness models. (R)

S4 File The nn_simulation.r program was used to simulate data for the normal-normal cost effectiveness models. (R)
